# Supplementary material for: Meta-regression to explain shrinkage and heterogeneity in large-scale replication projects
Source: PLoS One. 2025 Aug 1;20(8):e0327799. doi: 10.1371/journal.pone.0327799 (PMC12316214; doi:10.1371/journal.pone.0327799)
Supplement: S1 File — It includes data descriptives and supplementary tables, figures and analyses. (PDF) [file pone.0327799.s001.pdf]

A Data analysis protocol for case study

A.1 Merging data from Altmejd et al. (2019) (31) with `ReplicationSuccess::RProjects`

(31) aimed to predict *successful replication* and *relative effect size* using machine learning models in two large-scale replication projects and two many-lab experiments, in psychology and economics. Their binary criterion for successful replication was defined as a replication with significant effect (p-value ≤ 0.05) in the same direction as the original study. The relative effect size, on the other hand, is the replication effect size divided by the original effect size. For our case study, we will use only the two replication projects in Psychology (RPP) and in Experimental Economics (RPEE). The data was downloaded from the Open Science Framework (OSF, osf.io/4fn73/). In total 69 original-replication study-pairs from the RPP and 18 from the RPEE are included in the data. Note that only original studies with an effect interpreted as significant by the original authors were included in (31).

The `R`-package `ReplicationSuccess` (40) provides functionalities for the design and the analysis of replication studies and contains, among other, the data from both large-scale replication projects analysed here (in the data-object `RProjects`). In the `R`-package, all effect estimates were Fisher-z-transformed to assume normality. This enabled the authors of the package to recompute two-sided p-values using that the effect estimates on the Fisher-z scale. For our case study the outcomes of all original-replication study-pairs should be on the same scale, which is why we prefer using this version of effect size and merged the data published by (31) (`Altmejd`) with the `ReplicationSuccess::RProjects` data. To merge both data sources we used the original author names (`authors.o` in `Altmejd` and `study` in `RProjects`). More specifically we matched the names of the first authors. In the few cases where more than one paper with the same first author was included, the name of the second author was used to match the pairs. This level of detail was sufficient to match all included study-pairs. One of the original studies however had two replications (`study == "P Bressan, D Stranieri"`). We were able to match the correct original-replication-study-pair using the replication sample size. However, to avoid any dependencies in our data we want to include only one replications per study. We therefore decided to exclude the online replication (as discussed in the replication report on the OSF, osf.io/7vriw).

Table A.1: Summary of the continuous covariates used in (31).

	Experimental Economics			Psychology		
Covariate	Average	Min.	Max.	Average	Min.	Max.
p-value (O)	0.018	<0.001	0.07	0.015	<0.001	0.073
Effect estimate on Fisher-z scale (O)	0.57	0.12	1.2	0.5	0.17	1.3
Effect estimate on Fisher-z scale (R)	0.31	-0.12	0.93	0.24	-0.48	1.6
Power (O)	0.85	0.31	1	0.75	0.42	1
Planned power (R)	0.93	0.31	1	0.85	0.25	1
O paper length (nb pages in journal formatting)	28	16	45	11	1	31
Nb citations (O)	80	7	310	84	6	340
Nb authors (O)	2.7	2	4	2.8	1	9
Nb authors (R)	3.3	2	5	2.5	1	10
Avg author citations (O)	1'700	40	8'900	3'000	180	17'000
Avg author citations (R)	2'600	130	6'100	430	18	3'100
Max author citations (O)	4'200	54	22'000	6'300	330	37'000
Max author citations (R)	7'500	260	18'000	970	35	9'700
Share male authors (O)	0.87	0.5	1	0.65	0	1
Share male authors (R)	0.84	0.33	1	0.49	0	1

A.2 Descriptive statistics, transformation and initial covariate selection

Next, we will present and describe all the covariates that could be included as candidate covariates in the meta-regression to explain heterogeneity. First, we investigate any missing values in the data. Indeed, 1 of the study-pairs has many of the covariates missing: `SJ Heine, EE Buchtel, A Norenzayan`. This observation is deleted and the complete cases are retained. Then, Tables A.1 and A.2 present summary statistics of all covariates present in the data set, depending on whether they are of continuous or categorical nature.

A.2.1 Continuous covariates

Some of the continuous variables described in Table A.1 are related to the design and the results, while the others mainly describe the author composition of the original and the replication study. The effect size of the original studies are on average larger than the one of the replications which suggests that there is at least some shrinkage. The outcome of the meta-regression is the difference between original and replication effect size and a positive difference suggests shrinkage of effect size. The p-values of all included original studies are small, as only those with effects described as significant by the original authors were included. Figure A.1 shows the distribution of all continuous variables. All five citation variables are skewed, and need to be log-transformed before being included as candidate covariates. Only the averages and not the maxima of the author citations (in original and replication) will be included as candidate variables because those The two proportions, of male authors in the original or replication study, are most often equal to 1. Indeed among all included study-pairs 43% have only male original authors, while for 60.5% the majority of the original authors are male. In general however, this variable is hard to interpret without knowing the total number of authors. Therefore we suggest to only include the share of male original or replication authors together with the total number of authors of either the replication or the original study.

The original p-values, the power and the original effect size estimate $\hat{\theta}_o$ combined with its standard error σ_o convey the same information.

The final decision on which of the continuous covariates are included as candidate covariates and which, if any, transformations are applied is summarised in Table A.3.

Table A.2: Summary of the categorical and binary covariates used in (31).

		Experimental Economics	Psychology
Covariate	Level	n (%)	n (%)
Discipline	Economics	18 (100%)	—
	Cognitive	—	34 (50%)
	Social	—	34 (50%)
Max seniority of authors (O)	Assistant/ociate Pr.	2 (11.1%)	9 (13.2%)
	Professor	16 (88.9%)	56 (82.4%)
	Researcher	—	3 (4.4%)
Max seniority of authors (R)	Professor	18 (100%)	13 (19.1%)
	Assistant/ociate Pr.	—	44 (64.7%)
	Researcher	—	11 (16.2%)
Type of compensation (O)	cash	18 (100%)	14 (20.6%)
	credit or mixed	—	50 (73.5%)
	nothing	—	4 (5.9%)
Type of compensation (R)	cash	18 (100%)	25 (36.8%)
	credit or mixed	—	39 (57.4%)
	nothing	—	4 (5.9%)
Type of subjects (O)	community	1 (5.6%)	5 (7.4%)
	students	17 (94.4%)	56 (82.4%)
	anyone	—	7 (10.3%)
Type of subjects (R)	students	18 (100%)	55 (80.9%)
	anyone	—	4 (5.9%)
	community	—	6 (8.8%)
	online	—	3 (4.4%)
Country of original experiment	Australia	1 (5.6%)	1 (1.5%)
	Austria	1 (5.6%)	—
	Germany	2 (11.1%)	3 (4.4%)
	Switzerland	2 (11.1%)	—
	United Kingdom	1 (5.6%)	6 (8.8%)
	United States	11 (61.1%)	47 (69.1%)
	Canada	—	2 (2.9%)
	France	—	1 (1.5%)
	Israel	—	4 (5.9%)
	Italy	—	1 (1.5%)
	Netherlands	—	3 (4.4%)
Country of replication experiment	Austria	5 (27.8%)	1 (1.5%)
	Germany	2 (11.1%)	11 (16.2%)
	Singapore	5 (27.8%)	—
	United Kingdom	2 (11.1%)	4 (5.9%)
	United States	4 (22.2%)	36 (52.9%)
	Australia	—	1 (1.5%)
	Canada	—	2 (2.9%)
	Hong Kong	—	1 (1.5%)
	Israel	—	1 (1.5%)
	Italy	—	2 (2.9%)
	Netherlands	—	8 (11.8%)
	Uruguay	—	1 (1.5%)
O&R same language	0	2 (11.1%)	26 (38.2%)
	1	16 (88.9%)	42 (61.8%)
O&R same country	0	12 (66.7%)	37 (54.4%)
	1	6 (33.3%)	31 (45.6%)
O&R conducted on/offline	1	18 (100%)	62 (91.2%)
	0	—	6 (8.8%)
O&R type of subjects	0	1 (5.6%)	10 (14.7%)
	1	17 (94.4%)	58 (85.3%)
Lab in the US (O)	0	7 (38.9%)	21 (30.9%)
	1	11 (61.1%)	47 (69.1%)
Lab in the US (R)	0	14 (77.8%)	32 (47.1%)
	1	4 (22.2%)	36 (52.9%)

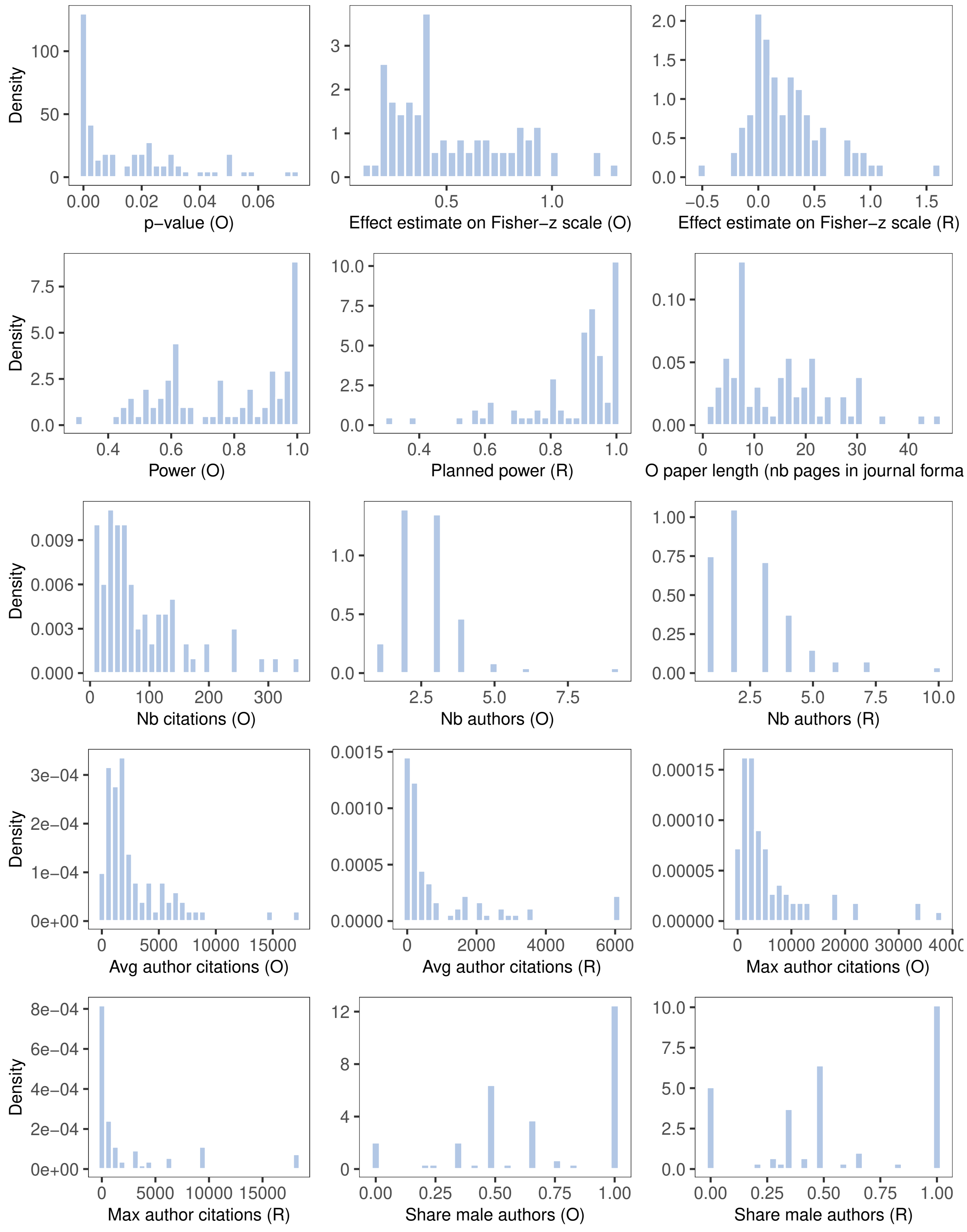

Figure A.1: Histograms of all continuous variables.

A.2.2 Categorical covariates

Table A.2 summarises the categorical covariates. To better understand the relationship between covariates and outcome (difference in effect size), we refer to the boxplots in Figure A.2. Note that, for readability of the Figure, the covariates country of original and replication experiment were already re-leveled to continent and the Assistant and Associate Professor

Covariate	Included as candidate variable	Transformation needed
p-value (O)	No	-
Effect estimate on Fisher-z scale (O)	No	-
Original standard error	Yes	σ_o
Effect estimate on Fisher-z scale (R)	No	-
Power (O)	No	-
O paper length (nb pages in journal formatting)	Yes	No
Nb citations (O)	Yes	log-transformation
Nb authors (O)	Yes	No
Nb authors (R)	Yes	No
Avg author citations (O)	Yes	log-transformation
Avg author citations (R)	Yes	log-transformation
Max author citations (O)	No	-
Max author citations (R)	No	-
Ratio authors male (O)	Yes	No, but only combined with 'Nb authors (O)'
Ratio authors male (R)	Yes	No, but only combined with 'Nb authors (R)'

Table A.3: Final decision for continuous covariates: will they be included as candidate variables, and if yes, which transformation is applied (if any).

levels of the seniority covariates were regrouped. Some of the categorical covariates will be adapted as described in Table A.4. Additionally the reference category and information on whether or not they are included as candidates in the meta-regression are specified in the Table. To reduce complexity summarising binary covariates, like "same country", are preferred over study specific factors with many levels, as for example "country of original experiment".

Covariate	Initial levels	Changed levels	Reference	Include
Discipline	Cognitive, Economics, Social	-	Cognitive	Yes
Max seniority of authors (O and R)	Assistant Professor, Professor, Associate Professor, Researcher	Assistant and Associate regrouped	Researcher	Yes for O, No for R
Type of compensation (O and R)	Cash, credit, mixed, nothing	-	Cash	No
Type of subject	Anyone, community, online, students	Students vs. all other levels	Students	No
Country of experiment (O and R)	Diverse countries	continents	Europe	No
Same language	1 = Yes and 0 = No	-	0 = No	Yes
Same country	1 = Yes and 0 = No	-	0 = No	Yes
Both conducted on/offline	1 = Yes and 0 = No	-	0 = No	No
Same type of subjects	1 = Yes and 0 = No	-	0 = No	Yes
Lab in the US (O)	1 = Yes and 0 = No	-	0 = No	No
Lab in the US (R)	1 = Yes and 0 = No	-	0 = No	No

Table A.4: Description of how the categorical covariates are releved and specification of the reference categories in addition to information on whether or not they are included as candidate variables.

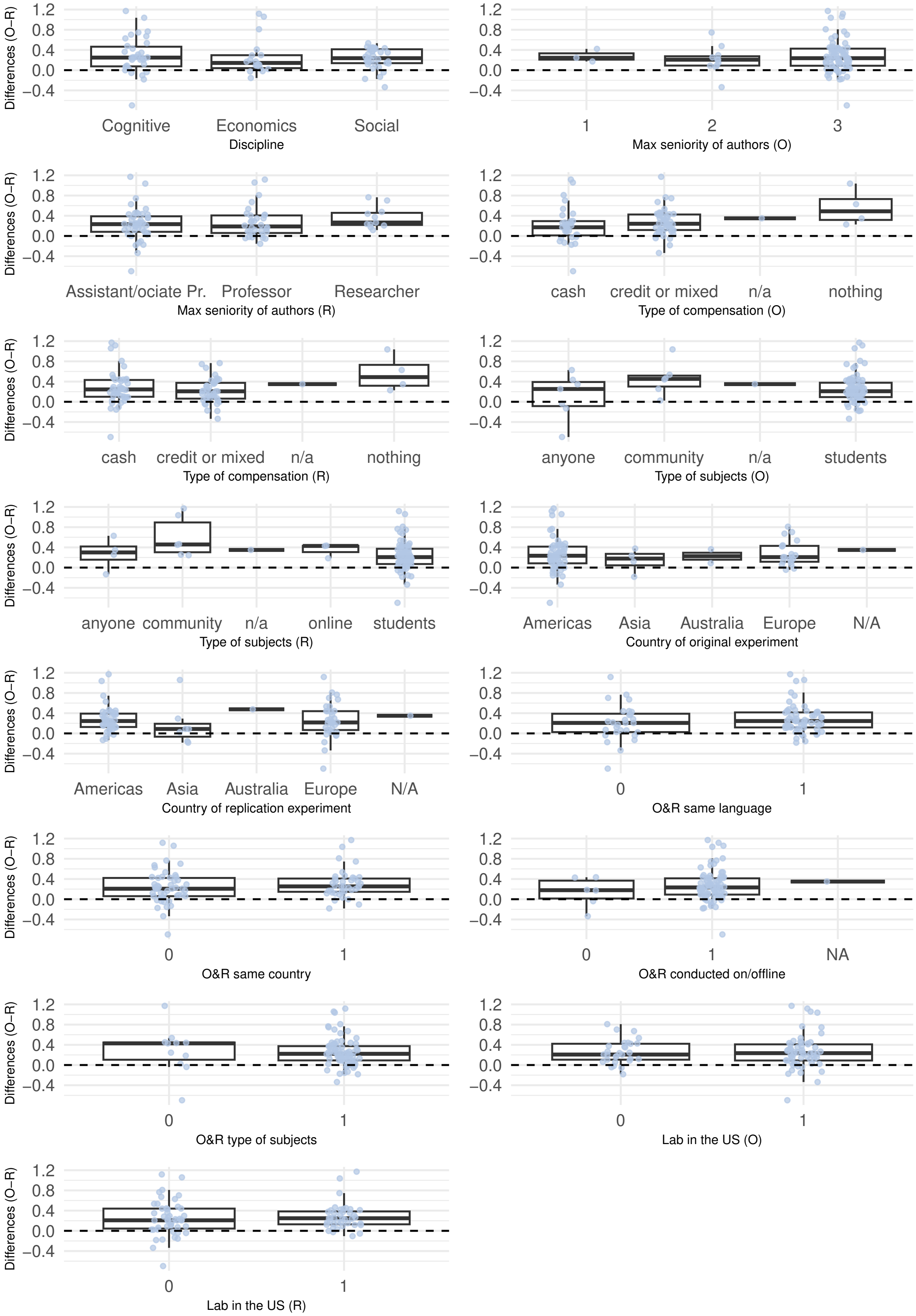

Figure A.2: Boxplots of all categorical covariates.

B Bubble plots - meta-regression models

Figure B.3 shows the association between the original standard error and the effect size differences, depending on whether the meta-regression uses an additive or a multiplicative version of the heterogeneity. The width of the intervals, especially the prediction intervals, are quite different.

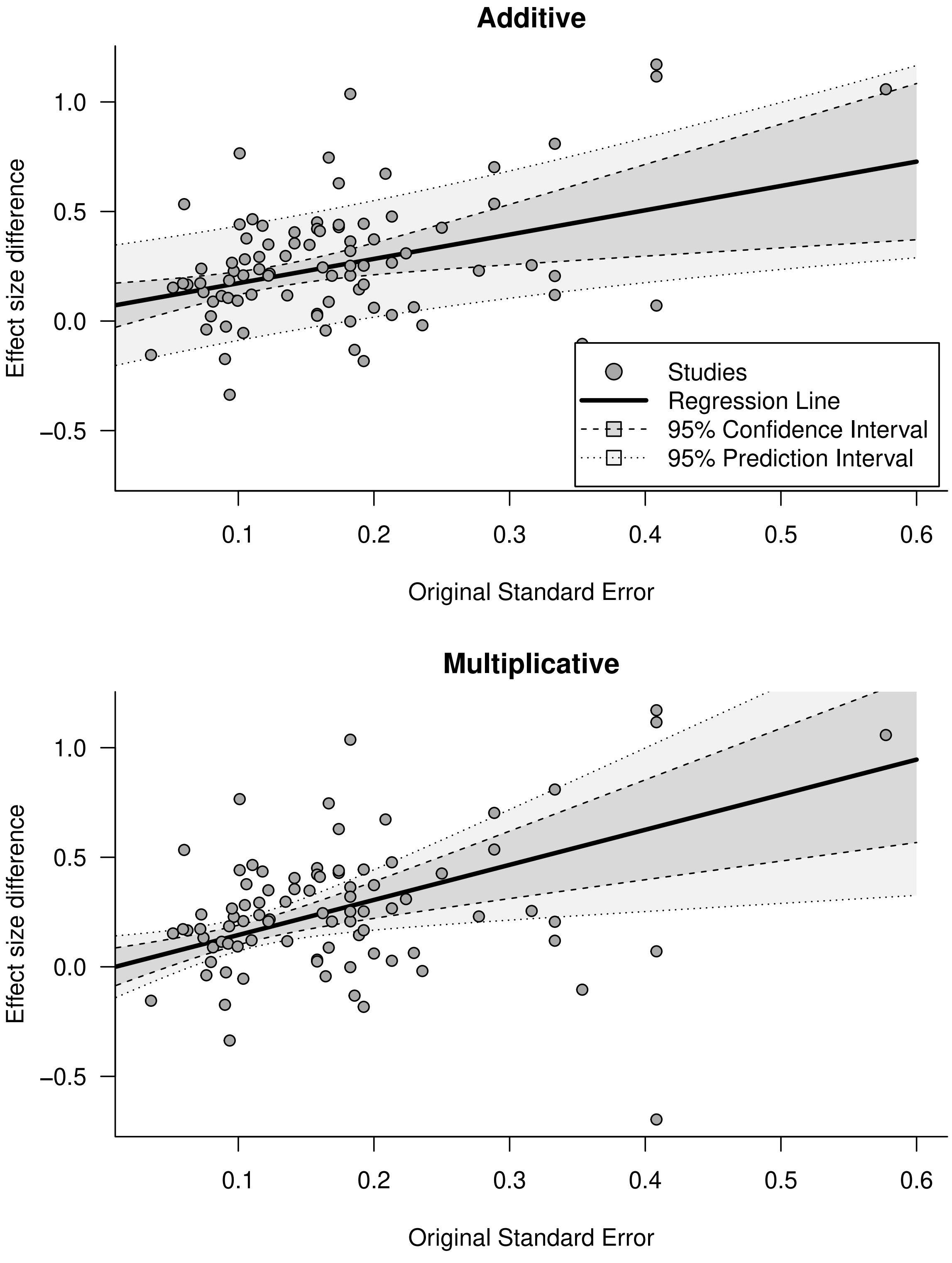

Figure B.3: Bubble plots showing the association between original standard error and the location, here the effect size difference. The underlying meta-regression models include only one location-covariate (original standard error) and no scale-covariate.

C Diagnostics of final location-scale models

Figure C.4 shows the normal QQ plots for the final additive and multiplicative heterogeneity model versions. The diagnostics shown are only for the finally selected location-scale meta-regression models. From these figures, we can conclude that the residuals are approximately normal.

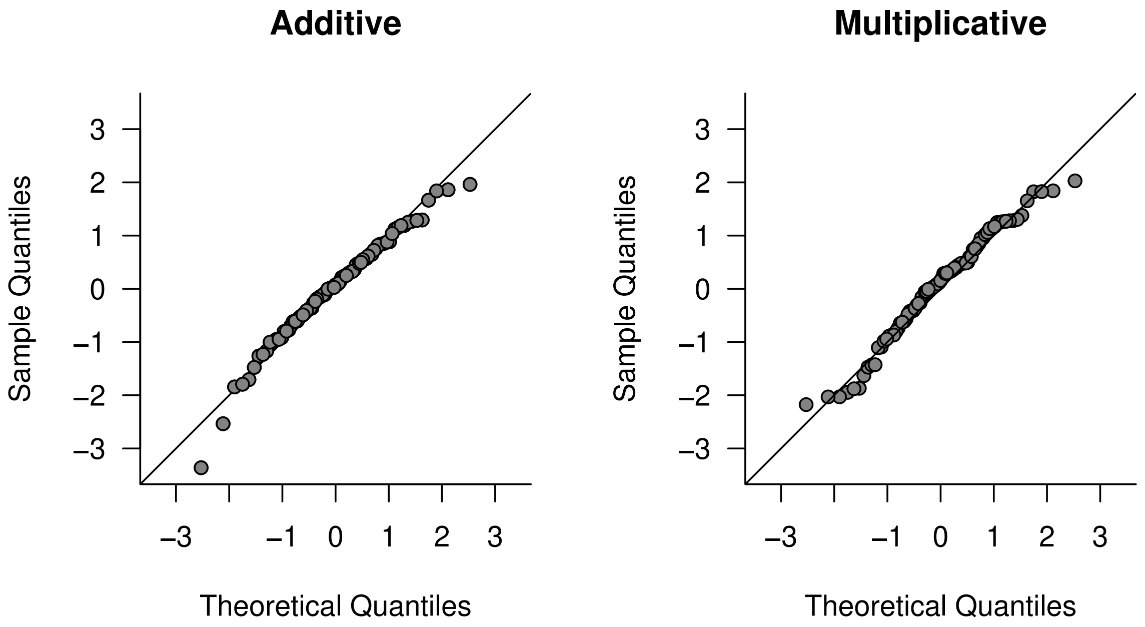

Figure C.4: Q-Q normal plot for the final location-scale model with additive and multiplicative heterogeneity.

D Model selection with Bayesian information criterion (BIC)

Figure D.5 shows the model selection using for both multiplicative and additive model versions, for meta-regressions modeling only the location. For the multiplicative version the model with four covariates would be selected, while for the additive model version, the one covariate, i.e., the original standard error, already minimizes the BIC. For the additive model we will continue the model selection process with three covariates, as the BIC of the model with three is only slightly larger, but this ensures that the residual heterogeneity $\tilde{\tau}^2$ is also reduced. Table D.5 shows the coefficients of the models resulting from the model selection based on BIC. Then, continuing the model selection, when selecting among the same covariates that were chosen for the location, now for the scale, we find the best ten models with respect to their BIC in Tables D.6 for the multiplicative version and D.7 for the additive version.

Table D.5: Summary of investigated location meta-regression models with multiplicative and additive heterogeneity where the final models are chosen based on their Bayesian information criterion. The location coefficient estimates are shown with their 95% confidence intervals and the residual heterogeneity. The first model is the weighted unadjuted meta-regression model relating the difference in effect size to a constant, as in Equations 3 and 4. For the second model, one covariate was added into the meta-regression as a proof-of-concept. The third model represents the final model selected via the model selection procedure with BIC.

	Unadjusted Model		Adjusted Model		Final Model	
	Estimate	95%CI	Estimate	95%CI	Estimate	95%CI
**Multiplicative**						
Intercept	0.16	0.11 to 0.20	-0.02	-0.11 to 0.08	0.01	-0.17 to 0.19
Original standard error	—	—	1.60	0.85 to 2.35	1.35	0.66 to 2.03
Nb authors (O)	—	—	—	—	0.04	0.00 to 0.07
O&R same country	—	—	—	—	0.10	0.02 to 0.18
Share male authors (O)	—	—	—	—	-0.21	-0.34 to -0.08
Heterogeneity	$\varphi = 2.004$	—	$\tilde{\varphi} = 1.682$	—	$\tilde{\varphi} = 1.359$	—
**Additive**						
Intercept	0.21	0.16 to 0.26	0.06	-0.05 to 0.17	0.09	-0.11 to 0.28
Original standard error	—	—	1.11	0.36 to 1.86	1.07	0.35 to 1.79
Nb authors (O)	—	—	—	—	0.04	0.00 to 0.08
Share male authors (O)	—	—	—	—	-0.19	-0.34 to -0.04
Heterogeneity	$\tau^2 = 0.021$	—	$\tilde{\tau}^2 = 0.017$	—	$\tilde{\tau}^2 = 0.012$	—

[1] Nb authors (O): number of authors on the original paper

[2] Discipline: Economics, Social or Coginitive Sciences, with reference being Cognitive Sciences

[3] Nb pages (O): number of pages of the original paper from the citation infromation

[4] O&R same language: Experiment of the original and replication study conducted in the same language

[5] Share male authors (O): proportion of male authors on the original author list

[6] Avg author citations (log, R): log-transformed average number of citations per author on the replication study

[7] Citations (log, O): log-transformed number of citations of the original study

Table D.6: The ten best multiplicative location-scale models according to their BIC and depending on the selection of location and scale covariates. The location and scale covariates are marked with a check-mark if they are present in the model and with a dash if they are not. The last row of the Table shows the BIC of the specific model.

	Model									
	1	2	3	4	5	6	7	8	9	10
**Location covariates**										
Original standard error	✓	✓	✓	✓	✓	✓	✓	✓	✓	✓
Nb authors (O)	✓	✓	✓	✓	✓	✓	✓	✓	✓	✓
O&R same country	-	-	-	-	-	-	-	-	-	-
Share male authors (O)	✓	✓	✓	-	✓	✓	✓	✓	✓	✓
**Scale covariates**										
Original standard error	-	-	-	-	-	-	-	-	-	-
Nb authors (O)	-	-	-	-	-	-	-	-	-	-
O&R same country	-	✓	✓	✓	-	-	✓	✓	✓	-
Share male authors (O)	-	-	-	-	-	-	-	-	-	-
BIC	21.28	21.62	22.28	22.31	22.61	22.72	22.85	22.92	22.96	23.30

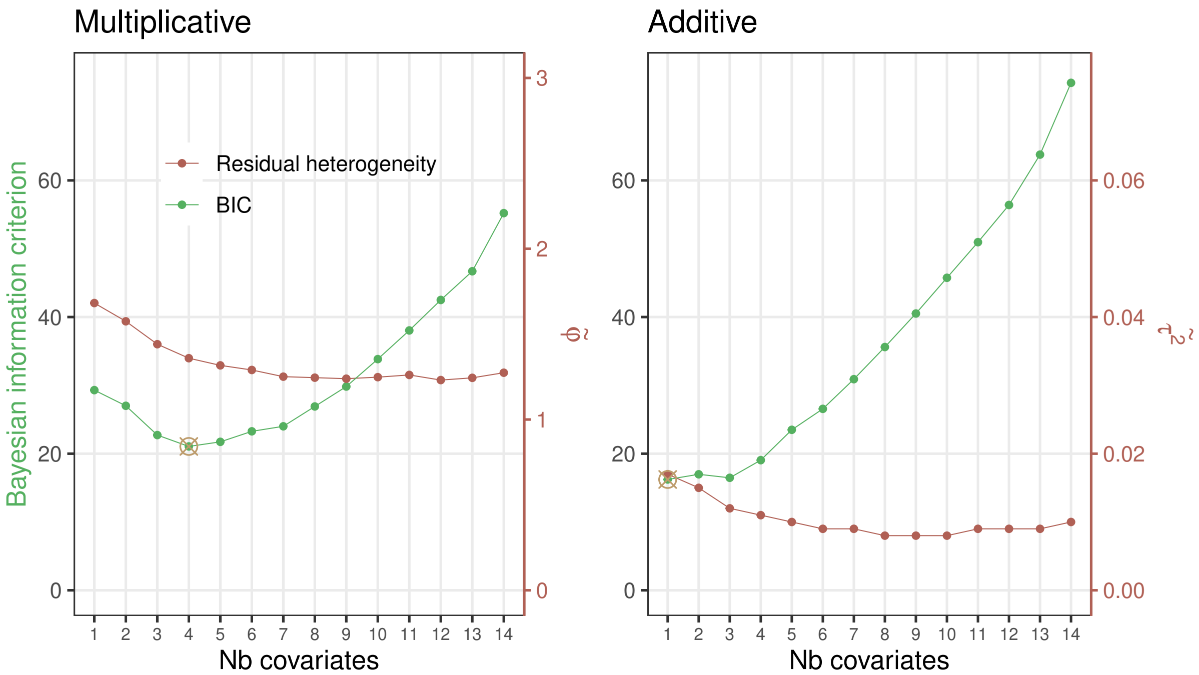

Figure D.5: The BIC for the multiplicative and the additive models with best performance (min BIC) for each possible number of covariates included. The residual multiplicative heterogeneity $\tilde{\varphi}$ and additive heterogeneity $\tilde{\tau}^2$ of the respective models are also shown. At least one covariate, namely the original standard error, is included. The minimum BIC value is highlighted.

Table D.7: The ten best additive location-scale models according to their BIC and depending on the selection of location and scale covariates. The location and scale covariates are marked with a check-mark if they are present in the model and with a dash if they are not. The last row of the Table shows the BIC of the specific model.

	Model									
	1	2	3	4	5	6	7	8	9	10
**Location covariates**										
Original standard error	✓	✓	✓	✓	✓	✓	✓	✓	✓	✓
Nb authors (O)	-	✓	✓	✓	-	✓	-	✓	✓	-
Share male authors (O)	-	✓	✓	-	-	-	-	✓	✓	-
**Scale covariates**										
Original standard error	-	-	✓	-	✓	✓	-	-	✓	✓
Nb authors (O)	-	-	-	-	-	-	✓	✓	✓	✓
Share male authors (O)	-	-	-	-	-	-	-	-	-	-
BIC	16.22	16.47	16.77	16.98	17.03	17.16	20.31	20.75	20.99	21.27
