# Supplementary material for: Meta-regression to explain shrinkage and heterogeneity in large-scale replication projects
Source: PLoS One. 2025 Aug 1;20(8):e0327799. doi: 10.1371/journal.pone.0327799 (PMC12316214; doi:10.1371/journal.pone.0327799)
Supplement: Attachment — Submitted filename: reviewer Report.docx [file pone.0327799.s002.docx]

The manuscript titled "Meta-regression to explain shrinkage and heterogeneity in large-scale replication projects", here is a professional reviewer report tailored to the essential criteria provided for reviewing a manuscript for PLOS ONE

1. Scientific Validity

The manuscript addresses a significant question in the field of meta-science and psychology the explanation of shrinkage and heterogeneity observed in large-scale replication projects. The research question is well-framed and aligns with current concerns in scientific replicability. The study employs meta-regression as the core analytical tool, which is appropriate given the dataset and research objectives. The inclusion of multiple replication projects enhances the generalizability of the conclusions. Statistical methods, including the use of the meta-regression with REML and appropriate control for collinearity, are technically sound. The authors also investigate the influence of sample size and publication year, which are sensible covariates. The conclusions are generally supported by the data; however, the authors should provide greater clarity on the robustness of their model assumptions and include sensitivity analyses to reinforce the interpretations of shrinkage effects.

2. Ethical Standards

As the manuscript relies solely on publicly available datasets from published studies, there are no new human or animal subjects involved. This aligns with standard ethical research practices. The manuscript, however, would benefit from a brief ethics statement clarifying the use of secondary data and confirming that no additional ethical approval was required.

3. Reporting Standards

The manuscript adheres reasonably well to reporting norms. However, it does not explicitly mention PRISMA or other reporting checklists, which would typically be expected in a meta-analytic framework. Including a PRISMA-style flow diagram summarizing inclusion criteria for studies and data points would enhance transparency. Furthermore, additional detail regarding study selection and any potential publication bias assessments would help align the reporting with community standards.

4. Data Availability

The authors have stated that all data are drawn from open-access replication projects. However, the Data Availability Statement should be more explicit, including direct links to the datasets used and clarifying how others can access the analytical code and results. Supplementary material such as code scripts or detailed appendices would be useful for reproducibility and transparency.

5. Technical Soundness

The methodology, centered on meta-regression analysis, is technically appropriate and explained with reasonable clarity. The authors appropriately model heterogeneity and assess predictor significance. However, more detail is needed on the handling of missing data, potential publication bias, and model diagnostics (e.g., residual plots, multicollinearity statistics). Including a sensitivity analysis or comparing multiple models could further substantiate their claims.

6. Language and Presentation

The manuscript is written in generally clear and professional English, though there are a few areas where language could be tightened for clarity and flow. For example, some statistical explanations could be simplified or supplemented with illustrative examples. Figures and tables are informative, but a few legends lack sufficient detail. All references appear complete and well-formatted.

7. Originality

The study offers a novel synthesis of existing replication project data to probe into the underexplored question of shrinkage patterns and heterogeneity explanations. While the topic has been touched upon in prior literature, the meta-regression approach applied across multiple datasets represents a unique and valuable contribution.

8. Scope of the Journal

The manuscript falls well within the multidisciplinary scope of PLOS ONE, particularly under the umbrella of social science methodology and statistical modeling in empirical research. It holds value for researchers across psychology, metascience, and statistics.

9. Reproducibility

The study is mostly reproducible in concept, but more explicit documentation of the analysis workflow, statistical software (e.g., R packages used), and scripts would significantly improve reproducibility. The authors are encouraged to provide a GitHub repository or OSF link with the complete replication materials.

10. Reviewer Conduct

This review has focused solely on the technical and methodological quality, clarity of presentation, and reproducibility of the study. Constructive feedback has been provided to assist the authors in enhancing their manuscript for publication.

Recommendation Minor Revisions

The manuscript is scientifically valuable and methodologically sound but would benefit from

- Inclusion of a PRISMA checklist or analogous reporting structure.
- A stronger Data Availability statement with links and code.
- Clarifications on model diagnostics and reproducibility materials.
- Minor language edits for clarity.

With these revisions, the manuscript would be suitable for publication in PLOS ONE.
